# Supplementary material for: SARS-CoV-2 Neutralizing Antibodies in Mexican Population: A Five Vaccine Comparison
Source: Diagnostics (Basel). 2023 Mar 22;13(6):1194. doi: 10.3390/diagnostics13061194 (PMC10046906; doi:10.3390/diagnostics13061194)
Supplement: Supplementary file 1 [file diagnostics-13-01194-s001.zip › diagnostics-2158786-supplementary.pdf]

Table S1. Average of inhibition level by time.

Average of inhibition level						
Months	Pfizer-BioNTech	CanSino	AstraZeneca	Sinovac	Moderna	Total/month
1		97.29	95.37		97.41	97.05
2	96.47				98.10	96.88
3	93.17	96.15	97.24	74.06		92.69
4				76.95		76.95
5	95.04		81.44	30.90		92.00
6	92.72	70.85	97.24		97.32	80.72
7	94.57	78.94	97.18			86.94
8	72.14			45.95		63.41
9	86.65					86.65
10	97.29					97.29
General	92.74	79.75	85.78	75.62	97.61	88.28

Table S2. Cross table of effectiveness.

Pfizer-BioNTech vs CanSino effectiveness

	Without inhibition	With inhibition	p
Pfizer-BioNTech	0	140	**0.001***
CanSino	6	43	

*Significance $p<0.05$ Chi-squared test
